# Pigs naturally exposed to *porcine circovirus* type 2 (PCV2) generate antibody responses capable to neutralise PCV2 isolates of different genotypes and geographic origins

**DOI:** 10.1186/1297-9716-45-29

**Published:** 2014-03-06

**Authors:** Sherry Kurtz, Llorenç Grau-Roma, Martí Cortey, Maria Fort, Fernando Rodríguez, Marina Sibila, Joaquim Segalés

**Affiliations:** 1 Centre de Recerca en Sanitat Animal (CReSA), UAB-IRTA, Campus de la Universitat Autònoma de Barcelona, 08193 Bellaterra, Barcelona, Spain; 2 Present address: Laboratory of Mycobacterial Diseases and Cellular Immunology, Division of Bacterial, Parasitic and Allergenic Products, Center for Biologics Evaluation and Research, U.S. Food and Drug Administration, Rockville, MD 20852, USA; 3 Departament de Sanitat i Anatomia Animals, Universitat Autònoma de Barcelona, 08193 Bellaterra, Barcelona, Spain; 4 Present address: School of Veterinary Medicine and Science, University of Nottingham, Sutton Bonington Campus, Leicestershire LE12 5RD, UK; 5 Present address: The Pirbright Institute, Ash Road, Woking GU24 0NF, UK; 6 Present address: Zoetis Manufacturing and Research, S.L., 17813 Vall de Bianya, Girona, Spain

## Abstract

*Porcine circovirus* type 2 (PCV2) is the essential infectious agent for PCV2-systemic disease (PCV2-SD, formerly known as postweaning multisystemic wasting syndrome) and other pathological conditions. Recent studies indicated antigenic variability amongst different PCV2 isolates and suggested that single amino acid changes within the capsid protein determine differences in the level of neutralization by specific monoclonal antibodies. The objective of the present study was to examine the cross-reactivity of PCV2 antibodies induced in the context of a natural infection against different PCV2 isolates belonging to genotypes PCV2a and PCV2b. Sera taken from several farms from animals of varying health status (PCV2-SD and age-matched healthy pigs and a set of slaughter-aged animals) were assayed for neutralizing activity against four PCV2 isolates from both predominant genotypes (PCV2a and PCV2b) and of differing geographic origins (Europe and North-America). Results showed that most of studied pigs (79 out of 82) contained neutralizing antibodies (NA) able to neutralize all four studied viral strains. Overall, pigs had significantly higher NA titres against PCV2a than against PCV2b (*P* < 0.001). Accordingly, studied serums were able to better neutralize Burgos390L4 and Stoon-1010 strains (PCV2a) than L-33-Sp-10-54 and MO/S-06 strains (PCV2b) (*P* < 0.001). No differences between capabilities of seroneutralization of viruses from different geographic origin were observed. Present data suggests that sequence differences between PCV2 isolates translate to functional antigenic differences in viral neutralization in vivo.

## Introduction

Porcine circoviruses (PCV) are small non-enveloped, single-stranded DNA viruses within the *Circoviridae* family. Within this family, there are two types of PCV, PCV type 1 (PCV1) and PCV type 2 (PCV2). PCV1 was firstly identified as a non-cytopathic contaminant of the porcine kidney cell line PK-15, but it does not apparently cause disease in swine [1,2]. PCV2 is the essential etiological agent of PCV2-systemic disease (PCV2-SD, formerly known as postweaning multi-systemic wasting disease, PMWS), as well as other porcine circovirus diseases (PCVD) [3-5]. Although firstly described in North America in 1991, PCV2-SD and PCV2 are now found in almost every pig-producing country in the world [6-8]. Animals suffering from PCV2-SD demonstrate clinical signs of wasting or decreased weight gain, as well as anaemia, diarrhoea and/or respiratory distress [7]. Due to increased mortality rates and the impact on weight gain, PCV2-SD has a serious economic impact on the swine production industry.

The PCV2 genome is typically 1767–1768 nucleotides in length and encodes three open reading frames (ORF): ORF1, which encodes the replicase proteins (*rep* and *rep*’), essential for viral replication; ORF2, encoding the capsid (Cap) protein, the structural protein of the virus; and ORF3, a protein that is not essential for viral replication, but might play a role in virulence [9-11]. PCV2 isolates can be classified phylogenetically into four genotypes, PCV2a, PCV2b, PCV2c and PCV2d [12], being the first one especially prevalent before the major outbreaks of disease worldwide, while the second one was mainly prevalent during and after major PCV2-SD epizootics as well as nowadays [12-17]. PCV2a and PCV2b show a relatively high level of nucleotide identity in the *rep* (97-100%) and *cap* genes (91-100%), and also high similarity at the amino acid level, where identity is 97-100% for Rep and 89-100% for Cap [18]. However, what the biologically relevant differences are between the phylogenetic groups and how those differences may impact virulence is still being determined.

In general, protective immunity against PCV2 is thought to depend on a strong humoral immune response [19-22], while little is known about the contribution of cell mediated immunity [23,24]. A protective humoral response is, in part, reliant on the production of neutralizing antibodies (NA) against the virus [20,21]. Several groups have undertaken studies to elucidate the immunorelevant epitopes within the viral proteins, particularly Cap [25-28]. These studies mapped several epitopes throughout the Cap protein, suggesting that the Cap itself is a major target of immune recognition, a fact that has been exploited in the production of several of the commercially licensed PCV2 vaccines [22,29]. Moreover, several groups have described monoclonal antibodies with neutralizing activity against PCV2 Cap, and have used these to map the dominant immunological epitopes important for neutralization within PCV1 and PCV2 Cap [26,27,30,31].

PCV2 vaccines seem to be able to induce a humoral immune response, characterized by producing NA that are cross-protective against both predominant PCV2 genotypes [22]. Cross-protection between PCV2a and PCV2b has been already demonstrated in an SPF pig infection model [32]. Studies done to map immunogenic epitopes in the PCV2 Cap protein have also demonstrated that there are several epitopes that are shared between PCV2 genotypes, as well as PCV1, which presents a basis for cross-neutralization [27,30]. Recent studies suggested that single amino acid changes within the Cap protein determined differences at the level of neutralization by monoclonal antibodies [30,33]. These results further support the evidence for antigenic variability. However, these studies describing antigenic variation have been done using monoclonal antibodies, which are specific for unique epitopes. Therefore, the objective of the present study was to expand previous works on PCV2 antibody neutralization by using a panel of polyclonal sera obtained from field studies to assess the level of cross-neutralization amongst different strains of PCV2, including both predominant genotypes from two different geographical regions (Europe and North-America).

## Materials and methods

### Cells

PCV1 and PCV2 negative PK-15 cells were grown in complete Dulbecco’s Modified Eagle Medium (DMEM), which was supplemented with 10% foetal bovine serum (FBS), PenStrep and L-glutamine (complete DMEM).

### Viruses

The PCV2 isolates used in this study were propagated in PCV-free PK-15 cells. Two isolates corresponded to genotype PCV2a: Burgos390L4 and Stoon-1010 strains were isolated from pigs in Spain and Canada [22], respectively. The other two were PCV2b genotype: MO/S-06 and L-33-Sp-10-54 strains isolated from pigs in United States [22] and Spain [34], respectively. In all cases, viruses were retrieved from lymph nodes of PCV2-SD affected pigs.

### Orf2/Cap sequence analysis and alignment

The complete *orf2* gene from each of the four PCV2 isolates used in the study was amplified and sequenced for sequence alignment and comparison using a previously described protocol [35]. Briefly, DNA was extracted from viral stocks using the QIAamp DNA Mini kit (Qiagen) according to manufacturer’s protocol. Resulting DNA was eluted in DNase, RNase free water. The PCV2 genome was amplified from each sample using two sets of PCR primers [35], and the PCR products were visualized on an agarose gel, followed by PCR product purification using the Geneclean Kit (Bio101). Both DNA strands were sequenced using sequencing primers and the automated ABI DNA Sequencer (Applied Biosystems). Percentages of nucleotide identities and sequence alignments were done using the Clone Manager 7 sequence analysis software (SciEd Central) and BioEdit Sequence Alignment Editor.

### Sera

Sera from twenty PCV2-SD affected animals aged between 11 and 21 weeks and seventeen age-matched healthy animals, coming both from six different Spanish PCV2-SD affected farms [36], collected during the years 2005–2006, were used in the study. In addition, 45 serum samples taken from animals at the time of slaughter, originating from nine additional farms without previously known clinical history, were also included in the study. The slaughterhouse sera were randomly selected from a serum sampling of healthy slaughter-age pigs in Spain in 2007 [37]. All sera came from non-PCV2 vaccinated pigs.

### Immunoperoxidase monolayer assay (IPMA)

The total PCV2-specific antibody titre in each serum was determined by immunoperoxidase monolayer assay (IPMA) using each of the four studied viruses strains as described previously [38]. Briefly, for each serum sample, sets of four fold dilutions were made (from 1:20 to 1:20 480) using complete DMEM in fixed 96 well plates containing PK-15 cells infected with one of the four tested viruses (L-33-Sp-10-54, MO/S-06, Burgos390L4 and Stoon-1010). The fixed cells were incubated with the diluted serum for 1 h at 37°C, after which the wells were washed using PBS with 0.05% Tween 20 (PBS Tw). To each well, 50 μL of protein A conjugated horseradish peroxidase (HRP) (Sigma) was added to a final concentration of 0.6 μg/mL in PBS Tween 20 (Tw), and the plates were incubated for a further 1 h at 37°C. Finally, the plates were washed with PBS Tw, and the HRP substrate solution of 3-amino-9-diethyl carbazole in 0.1 M acetate buffer with 0.05% hydrogen peroxide was added at 50 μL/well to reveal the reaction.

### Virus Neutralization Assay (VNA)

All sera samples were tested for their ability to neutralize each of the four test viruses (Stoon-1010, L-33-Sp-10-54, Burgos390L4, MO/S-06), in an in vitro cross neutralization assay. The cross neutralization assay was performed as described previously [21], with some modifications. Briefly, 50 μL of each, non-inactivated test serum was added to the first well in a row of a 96-well plate. The serum was diluted two-fold in from 1:2 to 1:4096 in the 96-well plate using complete DMEM. Each serum was tested in duplicate for each virus. Each PCV2 isolate was diluted in complete DMEM to obtain approximately 200 TCID_50_/well, and 50 μL of this dilution was added to each well of the 96-well plate. The plates were incubated at 37°C for 1 h. After incubation, 2 × 10^4^ freshly trypsinized PK15 cells in a 100 μL total volume were added to each well of the 96-well plate. The wells were mixed by pipetting, and the plates were incubated for 72 h at 37°C with 5% CO_2_. Each 96-well plate also contained a negative control row containing no serum, and a positive control row containing an antibody with known NA titre. After 72 h, the supernatant was removed, the wells washed one time with PBS containing 0.05% Tween 20, and the plates were fixed by the addition of cold absolute ethanol, and stored at −20°C. The rest of the procedure was equivalent to that indicated for the IPMA technique, as described above. The viral inoculum used for each assay was titrated to determine the accuracy of the viral concentration. For each well, the number of positive cells was enumerated using a microscope at 100× by counting those cells which displayed nuclear, cytoplasmic, or nuclear and cytoplasmic staining. Three fields from each well were examined. The percentage of virus neutralization (%VN) at each serum dilution was calculated using the following formula: %VN [1− (mean # of positive cells of the two replicas of each serum dilution/mean # of positive cells in the negative control wells)] × 100. The plates were also validated as described previously [21]. When the plates had been validated, the NA titre was calculated as the reciprocal of the last dilution for which a given serum sample was able to reduce the number of PCV2 infected cells by 50%. This value was designated as VNT50, and was reported as the neutralizing titre.

### Statistics

The total PCV2 IPMA antibody titres were codified from 0 to 6 using the four fold dilutions (from 1:20 to 1:20 480); i.e. an undiluted sample was codified as 0, a dilution factor of 1:20 was codified as 1, and so on. The virus neutralizing titres were directly used taking values from 0 (< 2) to 12 (> 4096). For every animal, four factors were considered: sample origin (Healthy, PCV2-SD or Slaughterhouse), PCV2 genotype (PCV2b or PCV2a), geographic origin of the virus strain (Europe or North-America) and each particular virus strain (L-33-Sp-10-54, MO/S-06, Burgos390L4 and Stoon-1010).

In order to determine the normality of the IPMA and VNA datasets, the Kolmogorov-Smirnov and the Shapiro-Wilk statistics were computed for every combination of factor and dataset (i.e. IPMA and Healthy animals). For those datasets that gave non-significant results (data following normality), the corresponding parametric analysis (1-factor ANOVA) was applied to test the existence of significant results between the different groups included in each factor. Conversely, the datasets that gave significant results in the normality tests (data not following normality), the corresponding non-parametric tests were computed: Mann Whitney’s *U* (one factor with two treatments) or Kruskal Wallis test (1 factor with more than two treatments).

Finally, the lineal correlation for every combination of treatment and factor between IPMA and VNA was also computed.

## Results

### *Cap* gene sequences and predicted amino acid sequences

A pairwise alignment comparing *cap* gene from each of the used viruses, at both the nucleic acid and amino acid levels was performed, the results of which are presented in Table 1. All the four viruses had a high identity at both nucleic and amino acid levels. Higher identity was observed between L-33-Sp-10-54 and MO/S-06 (% nucleic acid/amino acid identity: 99/99) followed between Burgos390L4 and Stoon-1010 (96/97), the first two belonging to genotype PCV2b and the last two to genotype PCV2a. Besides, higher divergences were observed when comparing strains belonging to different genotypes (92-93/90-92).

**Table 1:** Pairwise nucleic acid/amino acid sequence comparisons for the PCV2 capsid gene

**Strain**	**Burgos390L4 (Genotype a)**	**Stoon-1010 (Genotype a)**	**L-33-Sp-10-54 (Genotype b)**	**MO/S-06 (Genotype b)**
**Burgos390L4 (Genotype a)**	-	96/97	93/90	93/91
**Stoon-1010 (Genotype a)**	96/97	-	92/92	92/92
**L-33-Sp-10-54 (Genotype b)**	93/90	92/92	-	99/99
**MO/S-06 (Genotype b)**	93/91	92/92	99/99	-

**Figure 1:**
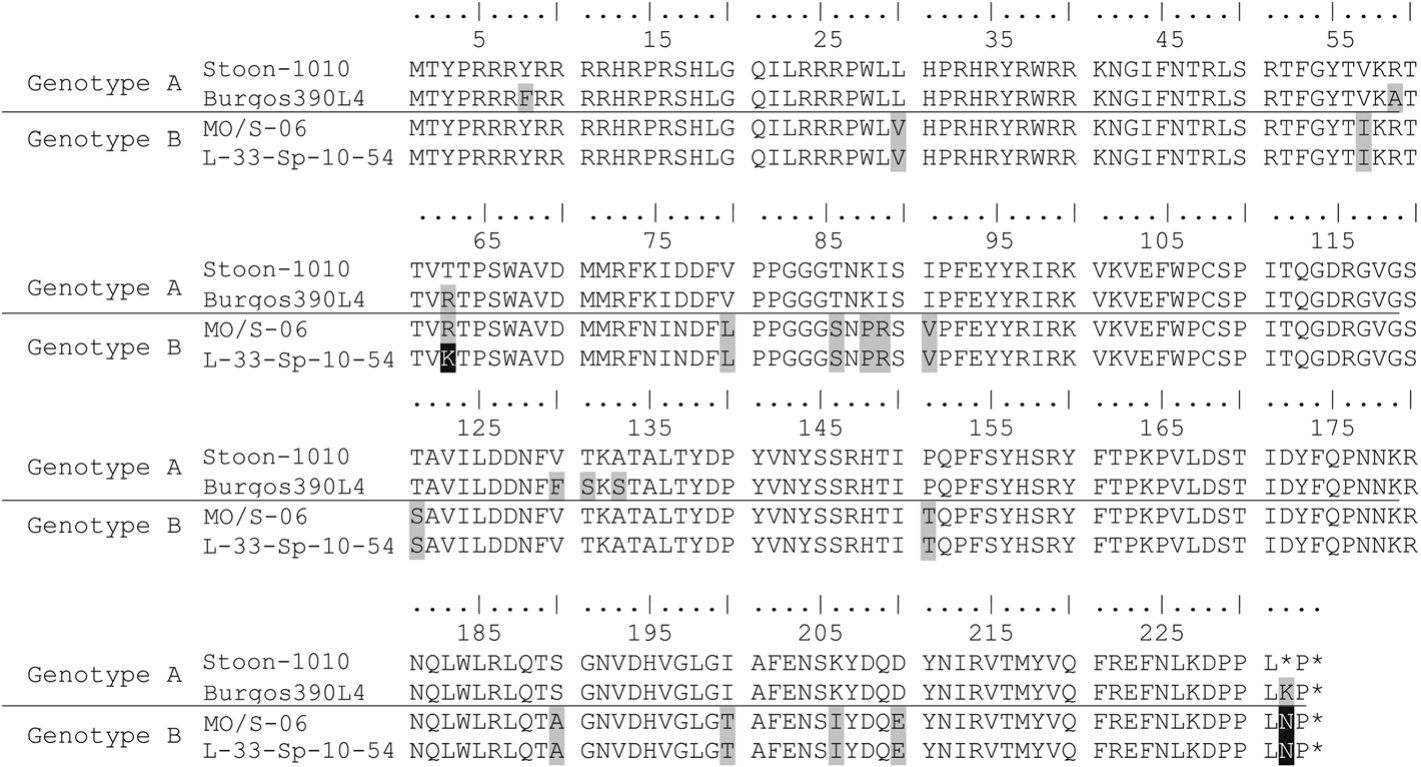
**PCV2 capsid amino acid sequence alignment of the four PCV2 isolates used for the cross-neutralization study.** The residues that differ from the genotype PCV2a Stoon-1010 strain are highlighted in grey and black.

The alignment of the PCV2 Cap amino acid sequences is displayed in Figure 1. Small number of amino acid differences occurred throughout the length of the protein, which are highlighted by shaded boxes (Figure 1). A total of 20 different amino acid positions showed differences amongst the studied strains. Fourteen out of these 20 positions showed differences only when comparing the strains belonging to different genotypes.

### IPMA and VNA

The neutralizing titres for each serum sample against each virus are presented in Table 2 (PCV2-SD and age-matched healthy pigs) and Table 3 (slaughterhouse animals). Seventy-nine out of the 82 studied sera including PCV2-SD affected animals, age-matched healthy counterparts, as well as slaughterhouse pigs, had titres of total (IPMA) and NA against all the four tested viruses. Only the serum of one animal, being a PCV2-SD-affected pig (reference number 9–51), had no PCV2 antibodies. Sera from two healthy pigs (reference numbers 8–48 and 10–41) contained NA against only 1 and 3 out of the 4 tested viruses, respectively.

**Table 2:** Neutralizing and IPMA titres for each PCV2-SD and age-matched healthy pig serum samples against each studied virus

**Identification data**			**Neutralizing titers**				**IPMA (Log**_**2**_**)**			
			**Genotype b**		**Genotype a**		**Genotype b**		**Genotype a**	
**Farm**	**Piglet**	**Classification**	**L-33-Sp-10-54 (EU)**	**MO/S-06 (NA)**	**Burgos390L4 (EU)**	**Stoon-1010 (NA)**	**L-33-Sp-10-54 (EU)**	**MO/S-06 (NA)**	**Burgos390L4 (EU)**	**Stoon-1010 (NA)**
4	10	Healthy	128	256	> 4096	512	14.32	14.32	14.32	14.32
4	71	Healthy	2048	128	1024	2048	14.32	14.32	14.32	14.32
4	45	Healthy	256	32	512	> 4096	14.32	14.32	10.32	14.32
4	62	Healthy	128	128	256	2048	14.32	14.32	14.32	12.32
6	7	Healthy	512	128	64	256	14.32	14.32	12.32	14.32
6	5	Healthy	32	64	64	32	8.32	6.32	4.32	6.32
6	93	Healthy	1024	256	64	512	14.32	14.32	14.32	14.32
8	48	Healthy	< 2	< 2	1024	< 2	14.32	14.32	14.32	14.32
8	42	Healthy	1024	256	512	2048	14.32	14.32	14.32	14.32
8	55	Healthy	64	256	2048	128	14.32	14.32	14.32	14.32
8	36	Healthy	512	128	1024	1024	14.32	14.32	14.32	14.32
9	3	Healthy	128	256	128	1048	14.32	14.32	14.32	14.32
9	9	Healthy	128	32	64	> 4096	14.32	14.32	14.32	14.32
9	20	Healthy	128	128	1024	1024	14.32	14.32	14.32	14.32
9	85	Healthy	128	128	1024	> 4096	14.32	14.32	14.32	14.32
10	41	Healthy	< 2	4	8	8	8.32	6.32	4.32	6.32
11	31	Healthy	8	4	8	8	10.32	6.32	6.32	6.32
4	75	PCV2-SD	128	32	16	64	8.32	6.32	6.32	6.32
4	64	PCV2-SD	32	16	16	3	14.32	14.32	14.32	14.32
6	36	PCV2-SD	> 4096	32	1024	256	14.32	10.32	10.32	10.32
6	14	PCV2-SD	32	26	16	3	8.32	6.32	6.32	6.32
6	64	PCV2-SD	256	64	512	28	14.32	14.32	12.32	14.32
8	90	PCV2-SD	64	8	16	4	12.32	10.32	10.32	10.32
8	82	PCV2-SD	128	128	1024	1024	14.32	14.32	14.32	14.32
9	4	PCV2-SD	4	8	16	4	10.32	8.32	6.32	6.32
9	18	PCV2-SD	16	32	1024	32	8.32	8.32	8.32	6.32
9	51	PCV2-SD	< 2	< 2	< 2	< 2	0	0	0	0
9	33	PCV2-SD	8	4	8	32	14.32	14.32	12.32	12.32
10	54	PCV2-SD	32	128	256	64	14.32	14.32	12.32	12.32
10	61	PCV2-SD	64	256	64	32	14.32	10.32	10.32	12.32
10	53	PCV2-SD	32	32	64	32	14.32	14.32	8.32	8.32
10	20	PCV2-SD	64	> 4096	64	128	14.32	14.32	12.32	12.32
10	93	PCV2-SD	256	128	1024	> 4096	14.32	14.32	10.32	14.32
11	63	PCV2-SD	64	128	1024	128	14.32	14.32	14.32	14.32
11	44	PCV2-SD	4	16	8	8	6.32	6.32	4.32	4.32
11	46	PCV2-SD	512	128	64	64	14.32	12.32	12.32	10.32
11	47	PCV2-SD	64	256	256	256	14.32	14.32	14.32	14.32

**Table 3:** Neutralizing and IPMA titres for each slaughterhouse pig serum samples against each studied virus

**Identification data**	**Neutralization titres**				**IPMA titres (Log**_**2**_**)**			
	**Genotype b**		**Genotype a**		**Genotype b**		**Genotype a**	
**Farm/pig**	**L-33-Sp-10-54 (EU)**	**MO/S-06 (NA)**	**Burgos390L4 (EU)**	**Stoon-1010 (NA)**	**L-33-Sp-10-54 (EU)**	**MO/S-06 (NA)**	**Burgos390L4 (EU)**	**Stoon-1010 (NA)**
A/1	128	16	> 4096	512	14.32	14.32	14.32	14.32
A/2	256	256	> 4096	2048	14.32	14.32	14.32	14.32
A/3	> 4096	128	> 4096	2048	14.32	14.32	12.32	12.32
A/4	> 4096	128	> 4096	> 4096	14.32	14.32	14.32	12.32
A/5	512	128	> 4096	> 4096	14.32	14.32	14.32	12.32
A/6	512	128	> 4096	> 4096	14.32	14.32	14.32	14.32
G2/A1	16	32	256	128	10.32	12.32	12.32	14.32
G2/A2	16	16	512	512	12.32	14.32	14.32	14.32
G2/A3	16	32	256	512	10.32	10.32	14.32	12.32
G2/A4	64	64	128	> 4096	12.32	10.32	12.32	14.32
G2/A5	32	64	64	> 4096	10.32	10.32	12.32	10.32
G9/A1	32	16	128	> 4096	8.32	8.32	8.32	10.32
G9/A2	128	32	256	> 4096	14.32	12.32	14.32	14.32
G9/A3	256	64	1024	> 4096	14.32	14.32	14.32	14.32
G9/A4	64	32	256	256	14.32	14.32	14.32	14.32
G9/A5	16	16	128	256	14.32	12.32	14.32	12.32
G16/A1	128	16	256	128	14.32	12.32	12.32	12.32
G16/A2	32	64	256	128	14.32	10.32	10.32	10.32
G16/A3	1024	32	256	256	14.32	10.32	14.32	14.32
G16/A4	1024	32	256	256	14.32	12.32	14.32	12.32
G16/A5	1024	32	512	256	12.32	8.32	8.32	8.32
G25/A1	256	32	2048	> 4096	14.32	14.32	14.32	14.32
G25/A2	256	32	2048	> 4096	14.32	14.32	14.32	14.32
G25/A3	256	512	> 4096	> 4096	14.32	14.32	14.32	14.32
G25/A4	256	128	256	128	14.32	14.32	12.32	14.32
G25/A5	256	> 4096	256	128	14.32	14.32	12.32	12.32
G27/A1	1024	32	256	128	14.32	14.32	12.32	14.32
G27/A2	32	16	32	> 4096	14.32	14.32	14.32	14.32
G27/A3	32	32	32	> 4096	14.32	14.32	14.32	14.32
G27/A5	64	64	256	2048	14.32	14.32	12.32	12.32
G49/A1	128	64	256	> 4096	14.32	14.32	14.32	14.32
G49/A2	256	512	1024	> 4096	14.32	14.32	14.32	14.32
G49/A3	1024	128	128	1024	14.32	14.32	14.32	14.32
G49/A4	1024	256	128	1024	14.32	14.32	14.32	14.32
G49/A5	1024	128	128	512	14.32	14.32	14.32	14.32
G73/A1	128	64	2048	512	14.32	14.32	14.32	14.32
G73/A2	128	32	2048	512	14.32	14.32	14.32	14.32
G73/A3	128	32	1024	256	14.32	14.32	14.32	14.32
G73/A4	2048	512	512	1024	14.32	14.32	14.32	14.32
G73/A5	> 4096	> 4096	512	1024	14.32	14.32	14.32	14.32
G87/A1	64	32	1024	512	14.32	14.32	12.32	10.32
G87/A2	64	32	2048	512	14.32	14.32	14.32	14.32
G87/A3	64	64	2048	256	14.32	14.32	14.32	14.32
G87/A4	64	64	> 4096	512	14.32	14.32	14.32	14.32
G87/A5	128	512	> 4096	512	14.32	14.32	14.32	14.32

**Figure 2:**
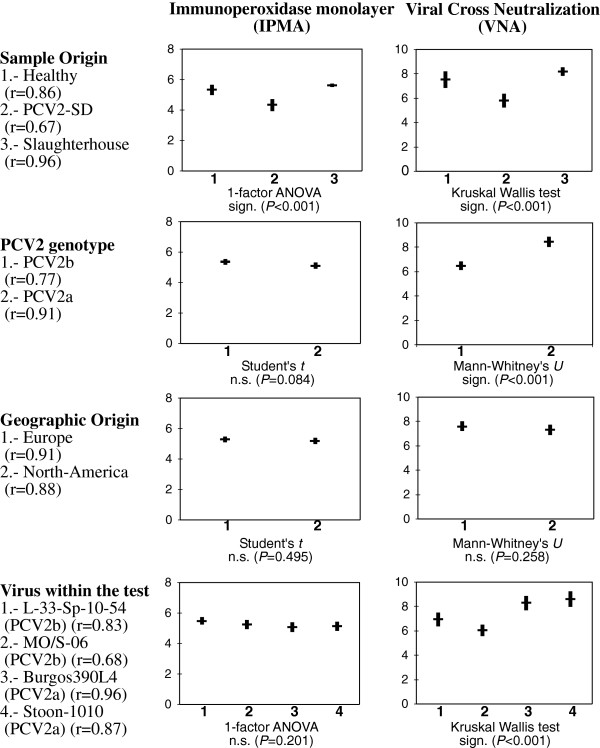
**Statistical analyses performed to the IPMA and VNA datasets: 95% confidence interval for the different treatments of the factors assayed (sample origin, PCV2 genotype, geographic origin and antibody).** Below each chart, the statistical test used and its significance are shown. After each treatment and within brackets, the linear correlation (r) between IPMA and VNA subsets is indicated. Vertical scales in the charts as described in the Material and methods section.

Results of the statistical analyses are summarized in Figure 2. Strong to very-strong correlations between IPMA titre and NA titre of the serum were detected in all the studied levels (r ranging from 0.67 to 0.96). However, IPMA and VNA gave different results. Thus, while VNA detected significant differences stemming from sample origin, the PCV2 genotype, and the virus strain used, the IPMA only detected significant differences in the sample origin. Both IPMA and VNA showed significantly lower antibody titres in PCV2-SD-affected pigs than in their age-matched healthy counterparts and slaughterhouse pigs (mean total PCV2-antibodies titres and neutralizing titres for healthy, PCV2-SD and slaughterhouse animals were 5.21, 4.38, 5.69 and 7.53, 5.80 and 8.17, respectively, *P* < 0.001). Globally, sera from naturally infected pigs showed significantly higher NA titres against PCV2a genotype viruses than against the PCV2b genotype (mean neutralizing titres for PCV2b and PCV2a were 6.12 and 8.44, respectively, *P* < 0.001). When analysing the specific virus strain, Burgos390L4 and Stoon-1010 (both belonging to genotype a) were better neutralized than L-33-Sp-10-54 and MO/S-6 (both belonging to genotype b) (*P* < 0.001). Finally, no statistical differences were found either in total antibody titre or in NA titres comparing viruses of different geographical origin (*P* = 0.495 and *P* = 0.258, respectively).

## Discussion

The present study attempted to investigate the role that sequence variability amongst PCV2 isolates from different origins and genotypes may play in their ability to be neutralized, giving a further role for antigenic variation in the immune response to PCV2. While there is a high degree of genomic similarity among the PCV2 isolates, as determined in this study and in previous ones [39], monoclonal antibodies have suggested the existence of antigenic differences within PCV2 that yield significant neutralization differences [25-27,30]. In a natural infection scenario, however, the situation is further more complex, since there are antibodies generated against multiple viral epitopes. Therefore, the present study sought to examine the presence of cross-neutralizing antibodies in the context of a natural infection. Sera taken from several farms from animals of varying health status (PCV2-SD and age-matched healthy pigs and a set of slaughter-aged animals) were assayed for neutralizing activity against PCV2 isolates from both predominant genotypes and of differing geographic origins.

Obtained results showed that most of studied pigs contained NA able to neutralize all four studied viral strains. Moreover, there was a significant positive correlation between total antibody titres and NA titres, which is in agreement with previous works [21,22]. Both IPMA and VNA showed lower titres of antibodies in PCV2-SD-affected pigs compared to their age-matched healthy counterparts and to slaughterhouse pigs. This is also in agreement with previous works [20,21] and supports the notion that PCV2-SD-affected pigs develop an impaired humoral response against PCV2.

A salient feature was that IPMA and VNA displayed different results when compared to the used virus strain and the genotype. Thus, meanwhile total antibody titres measured by means of IPMA in the studied sera did not show differences in this regards, higher NA titres were detected against PCV2a isolates than against PCV2b ones. Accordingly, studied sera were able to neutralize Burgos390L4 and Stoon-1010 strains (PCV2a) better than L-33-Sp-10-54 and MO/S-06 strains (PCV2b). These results support the hypothesis that there is a difference in the antigenic properties of these viruses [26-28,30,33], results that seem to be confirmed after the sequence analysis of the 4 PCV2 isolates used in this assay for the in vitro studies. To date, several specific amino acid positions have been indicated as important for PCV2 neutralization: 59 [28], 131, 151, 190, 191 [33] and the C-terminal area including residues 231–233 [27]. Looking back at the analysis of the amino acid sequence differences among the studied viruses, it was observed that 5 out of these 6 positions (59, 131, 151, 190, and 231–233) showed variations among the studied viruses. Among them, positions 151,190 and 231–233 allowed discrimination of both genotypes, while variations in positions 59 and 131 were only observed in Burgos390L4 strain (PCV2a). On one hand, mutation of T → A at position 190 has been suggested to cause loss of neutralization capabilities [33]. Interestingly, both strains belonging to PCV2b, which were less effectively neutralized, contained A at position 190, while both PCV2a strains had S in this position. In the same line, position 59 has been suggested to be a critical position for PCV2a seroneutralization, and the change from A to R was indicated to cause loss of the neutralizing capacity [28]. Amongst the studied strains, this change was observed in both strains belonging to PCV2b, as well as in Stoon-1010 (PCV2a). In addition, Saha et al. also indicated that mutations the change of P → T at position 151 resulted in an increase of neutralization [33]. This is in contrast with the present results, in which amino acids P and T were present in PCV2a and PCV2b, respectively. Finally, Saha et al. also suggested that a mutation of T → P in position 131 reduced neutralization, while all the here presented strains except Burgos390L4 contained a T in this position [33]. Taken together, results of the present study support the idea that described amino acid positions may have importance in PCV2 seroneutralization capacity. However, results also point towards the idea that there is no a single epitope responsible for the susceptibility of the different PCV2 strain to seroneutralization. Likely, the overall neutralization capability of a serum depends on the balance between existing antibodies against the different neutralizing epitopes.

The group of serum samples coming from PCV2-SD affected farms was previously included in a sequencing study which showed that, in these farms, the vast majority of recovered PCV2 sequences belonged to PCV2b genotype [40]. Besides, attempts to sequencing PCV2 from the serum samples coming from the slaughterhouse were not performed, since it is unlikely to detect the presence of PCV2 at this age [41,42]. Nevertheless, similarly to what was observed in our PCV2-SD affected farms [40], several field studies have demonstrated that PCV2b genotype is currently predominant in the field [12,14,43]. Moreover, those studies have described a shift of infection prevalence from PCV2a to PCV2b [12,14,43]. The here observed lower ability of the field serum samples to neutralize viruses belonging to PCV2b than PCV2a might have facilitated the spread of the first over the second, which could have facilitated the occurrence of the reported shift. The reasons why sera coming from pigs with predominance of PCV2b infection showed lower ability to neutralize in vitro viruses belonging to PCV2b than PCV2a could not be determined in this study. It cannot be ruled out that PCV2a circulated also among the studied pigs. Hypothetical PCV2a circulation at earlier ages and/or a lower PCV2a loads than those of PCV2b might have caused the lack (or minimal) of PCV2a detection. The study of serum samples coming from experimental infections with PCV2a, PCV2b and PCV2a/PCV2b should help in clarifying these hypotheses.

Present data also showed no differences between capacity of seroneutralization of viruses of different geographic origin (Europe and North America), highlighting that viral genetic differences but not their geographic origin may determine different magnitudes of humoral response [22]. Further neutralization studies including other PCV2 strains belonging to different genogroups within genotypes should contribute to understand the role of PCV2 genetic variation with the capacity of generation of NA.

Of particular importance is how these antigenic differences may relate to the production of a vaccine that would protect against all PCV2 isolates. Cross-protection between PCV2 genotypes has been demonstrated experimentally [22,32] and is further supported by the efficacy of PCV2a-based vaccines under field [8] and experimental [22] conditions. Nevertheless, a recent experimental study indicated that a PCV2 vaccine based on genotype 2b was more effective in protecting pigs against the effects of PCV2b or concurrent PCV2a–PCV2b challenge compared to a vaccine based on the PCV2a genotype [44]. Since protection against PCV2 is known to be dependent on the generation of NA [20,21], presented data showing differential ability of sera to neutralize viruses of different genotypes may explain the hypothesis that PCV2a vaccines might be less effective in reducing the prevalence of PCV2b than PCV2a [44]. The present study suggests that, in the next future, may be worthwhile to attempt to create vaccines that more specifically target the PCV2b and/or PCV2b/PCV2a antigenic epitopes to boost the degree of vaccine efficacy across all genotypes.

In summary, results of the present work suggests that sequence differences between PCV2 isolates translate to functional antigenic differences in viral neutralization in vivo, which are related to the virus genotype but not with its potential geographic origin.

## Competing interests

The authors declare that they have no competing interests.

## Authors’ contributions

Conceived and designed the experiments: SK, FR, JS. Performed the experiments: SK, LGR, MS. Analysed the data: SK, LGR, MC, MF, JS. Wrote the paper: SK, LGR, JS. All authors read and approved the final manuscript.
